# Amino alkyl-alkyl/aryl sulphides (DRDE-07 and analogues) as promising cytoprotectants for sulphur and nitrogen mustards – A review

**DOI:** 10.1016/j.toxrep.2025.102175

**Published:** 2025-11-24

**Authors:** R. Vijayaraghavan, M. Sharma

**Affiliations:** a Defence Research and Development Establishment (DRDE), Gwalior 474002, India; b Department of Research and Development, Saveetha Institute of Medical and Technical Sciences, Chennai 602105, India; c Professor, School of Studies in Pharmaceutical Sciences, Jiwaji University, Gwalior 474011, India

**Keywords:** Sulphur mustard, Nitrogen mustard, Amifostine, DRDE-07, DRDE-30, DRDE-35, Cytoprotectant

## Abstract

Several antidotes and medical countermeasures were experimented for the protection of one of the notorious chemical warfare agents, the mustard gas, also known as sulphur mustard (SM). Among them, the DRDE series of compounds (amino alkyl-alkyl/aryl sulphides) showed good protection and safety in pre-clinical studies as a prophylactic antidote when administered orally. These compounds showed better protection compared to other experimented molecules, such as amifostine, N-acetylcysteine, melatonin, sodium thiosulphate, and flavonoids. Although many studies have focused on the DRDE-07 molecule, the structurally related DRDE-30 and DRDE-35 have shown better safety and protection. Among them, DRDE-30 showed the best protection for SM and nitrogen mustards, as well as against radiation, and also as a cytoprotectant for anticancer agents. In this review, all the published papers on the DRDE series of compounds are compiled and discussed, with the aim that if any one of these molecules is recommended as an oral prophylactic drug, further development would occur for a more effective, broad-spectrum cytoprotectant.

## Highlights

• Sulphur and nitrogen mustards are alkylating agents that cause systemic toxicity.
• Percutaneous administration in animal models induces oxidative stress.
• DNA damage, decreased glutathione, and increased malondialdehyde are the effects.
• A satisfactory antidote has not yet been identified.
• Amino alkyl-alkyl/aryl sulphides (DRDE-07 series) protected prophylactically.

## Graphical Abstract

**Figure:**
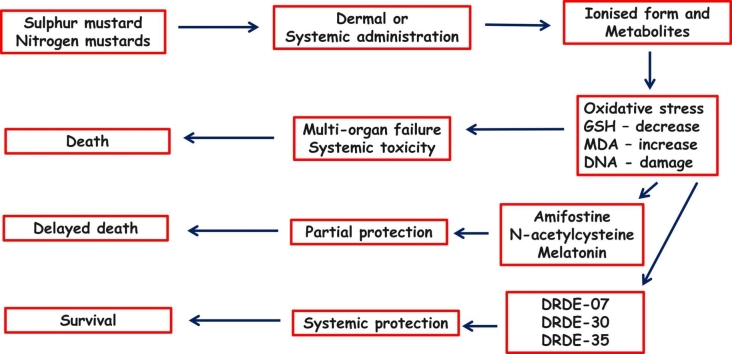

## 1 Introduction

World Wars I and II, and the later years, witnessed the use of chemical, biological and nuclear weapons as mass casualty agents. Although the agents are common materials, due to the severe morbidity and mortality that they could inflict, they were modified and diverted in the war scenario. Among them, mustard gas, known as sulphur mustard (SM), chemically bis(2-chloroethyl) sulphide, is an extremely toxic and incapacitating molecule [1]. SM is a bifunctional alkylating agent and, on physical contact, causes serious blisters on human skin [2], [3]. Several reports are available on the use of SM as a chemical warfare agent [4], [5]. SM is a well-studied cytotoxic, mutagenic and carcinogenic agent [6]. SM can rapidly enter dermal, inhalation and oral routes and, in contact with biological materials, rearranges to form an extremely reactive ethylene episulphonium ion [7]. This alkylates all micro and macromolecules, and particularly forms monofunctional and bifunctional intra- and inter-strand cross-links with DNA [2], [3]. The skin, eyes and the respiratory system are the main targets for SM. Due to extreme reactivity, SM causes serious systemic toxicity, and death occurs progressively. Even when administered via the percutaneous route in laboratory animals, SM shows extreme lethality [8.], [9]. Several mechanisms have been proposed, viz., poly (ADP-ribose) polymerase activation, NAD^+^ depletion, inhibition of glycolysis and DNA damage, for the skin blisters as well as for the systemic toxicity of SM [5], [10].

Though the Chemical Weapons Convention (CWC) has successfully eradicated all the stockpiled chemical warfare agents, the clandestine use of SM still exists during war and possibly by terrorist groups [11]. For this purpose, an antidote that can be used prophylactically or therapeutically would be useful. Moreover, such an important molecule, which can also be used for nitrogen mustards (HN-1, HN-2 [mechlorethamine], and HN-3), as well as a radioprotector, and as a cytoprotectant during cancer chemotherapy, would be an ideal choice [12]. One such molecule, amifostine (also known as W*R*-2721; S-2-[3-aminopropylamino]ethyl phosphorothioate), developed by the U.S. Army's Drug Synthesis and Development Programme, has been used as a cytoprotectant and is effective when administered by the parenteral route [13], [14]. Many compounds related to amifostine with better bioavailability and also orally effective of the series of amino alkyl-alkyl/aryl sulphides (DRDE analogues) were synthesised and pre-clinical studies were carried out proving the efficacy and safety [15]. These compounds showed better prophylactic protection compared to other experimented molecules. The code DRDE stands for the Defence Research and Development Establishment (Gwalior, India), one of the laboratories of the Defence Research and Development Organisation (DRDO, India). Hence, the purpose of this review is to compile all publications on DRDE analogues from all indexed sources for further development. The compounds were effective against sulphur and nitrogen mustards, as well as a radioprotector and a cytoprotectant for anticancer agents.

## 2 Cytotoxic effects of mustard compounds

SM administered percutaneously affects the body weight seriously, with a progressive decrease. All the exposed animals (mice and rats) die slowly over a period of two weeks. The LD_50_ of SM and nitrogen mustards in laboratory animals, administered via various routes, is shown in Table 1. Interestingly, percutaneous administration is more toxic than the subcutaneous route in animal models. The vital organs are also affected, and a decrease in spleen weight is observed. Significant decrease in the white blood cell (WBC) count and an increase in serum transaminases and alkaline phosphatases (ALPs) are observed. A significant decrease in reduced (GSH) and oxidised glutathione (GSSG) and an increase in thiobarbituric acid reactive substance and DNA fragmentation are also observed [16], [17]. The nitrogen mustards (HN-1, HN-2 and HN-3) also show similar effects [17]. Percutaneous administrations of SM and nitrogen mustards cause systemic toxicity and affect all vital organs, and show histopathological lesions [18], [19].

**Table 1:** Acute toxicity of mustard compounds.

S.No.	Agent	Route	Animal	LD_50_
1	SM	p.c.	Mice	8.1
		s.c.	Mice	23.0
		p.c.	Rat	2.4
		s.c.	Rat	3.4
		p.c.	Rabbit	1.0
		s.c.	Rabbit	80.0
2	HN-1	p.c.	Mice	16.8
		s.c.	Mice	2.5
3	HN-2	p.c.	Mice	33.6
		s.c.	Mice	5.0
4	HN-3	p.c.	Mice	23.8
		s.c.	Mice	4.8
		p.c.	Rabbit	4.9
		s.c.	Rabbit	16.3

SM = sulphur mustard

HN = nitrogen mustard

p.c. = percutaneous

s.c. = subcutaneous

Data sourced from [9], [18], [33]

Many anticancer agents, as well as radiation, exhibit similar effects in animal models and in humans [20], [21], [22], [23].

## 3 Acute toxicity studies of DRDE series compounds

**Fig. 1:**
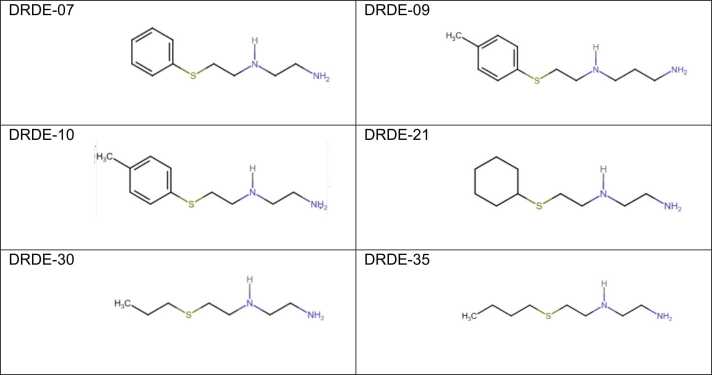
Chemical structure of the DRDE-07 series compounds.

The structures, molecular weights and acute toxicity studies of some of the effective compounds are given in Table 2. The chemical structures of the molecules are shown in Fig. 1. Among the compounds, DRDE-30 (S-2(2-aminoethyl amino) ethyl propyl sulphide) was found to be the least toxic compared to the other analogues in mice, viz., DRDE-07 (S-2[2-aminoethylamino] ethyl phenyl sulphide), DRDE-10 (S-2[2-aminoethylamino] ethyl tolyl sulphide) and DRDE-35 (S-2(2-aminoethyl amino) ethyl butyl sulphide). Only DRDE-07 was tested in mice and rats by oral and intraperitoneal routes. The reported LD_50_ of amifostine in mice is 1049 mg/kg (p.o.) [24].

**Table 2:** Acute LD_50_ of DRDE-07 and the analogues.

S.No.	Code	Structure	Mol.wt	Mice		Rat		Rabbit
				p.o.	i.p	p.o.	i.p.	p.o.
1	DRDE-07	NH_2_-(CH_2_)_2_-NH-(CH_2_)_2_-S-C_6_H_5_	287	1247	336	1599	141	> 300
2	DRDE-09	NH_2_-(CH_2_)_3_-NH-(CH_2_)_2_-S-C_6_H_4_-CH_3_	315	1131	-	> 300	-	> 300
3	DRDE-10	NH_2_-(CH_2_)_2_-NH-(CH_2_)_2_-S-C_6_H_4_-CH_3_	301	1902	-	> 300	-	> 300
4	DRDE-21	NH_2_-(CH_2_)_2_-NH-(CH_2_)_2_-S-C_6_H_11_	293	1131	-	> 300	-	> 300
5	DRDE-30	NH_2_-(CH_2_)_2_-NH-(CH_2_)_2_-S-(CH_2_)_2_-CH_3_	253	4525	-	> 300	-	> 300
6	DRDE-35	NH_2_-(CH_2_)_2_-NH-(CH_2_)_2_-S-(CH_2_)_3_-CH_3_	267	2262	-	> 300	-	> 300

All compounds are 2HCl.2H_2_O

Mol.wt = Molecular weight

LD_50_ is based on a single dose and a 14-day observation period expressed as mg/kg.

> 300 mg/kg, the dose was used for protection studies. LD_50_ was not determined.

p.o. = per oral; i.p. = intraperitoneal.

data sourced from [9], [15], [16], [36], [57]

## 4 Effect of DRDE-07 analogues on organ systems

### 4.1 Analgesic and anti-inflammatory activity of the analogues in mice

For analgesic activity, the acetic acid-induced writhing test and the formalin-induced paw licking were done. The persistent pain model of formalin-induced hind paw licking was carried out to test the effect of the compounds on neurogenic pain and peripheral pain (early and late phases). For acute inflammation, carrageenan-induced hind paw oedema was carried out. DRDE-07 and DRDE-30 showed a significant reduction (∼80 %) in the acetic acid-induced writhing test. DRDE-07 and DRDE-30 showed very good (∼80 %), while DRDE-35 showed moderate (∼60 %) reduction in the second and late phases of formalin-induced paw licking. All the analogues showed a significant reduction in carrageenan-induced paw oedema (∼60 %). The analgesic and anti-inflammatory activities of the antidotes were comparable to those of aspirin [25]. The anti-inflammatory activity of DRDE-35 in the lipopolysaccharide (LPS) induced mouse peritonitis model was also proved. DRDE-35 has been shown to decrease local and systemic inflammatory mediators, including cytokines and chemokines [26].

### 4.2 Effect of DRDE-07 on cardio-vascular system in anesthetised rats

The effect of DRDE-07 was studied on anesthetised rats by oral and intraperitoneal administration. Mean arterial blood pressure, heart rate, respiratory rate and tidal volume were monitored on an oscillograph. Intraperitoneal administration of 0.5 and 1.0 LD_50_ and oral administration of 0.25 LD_50_ did not show any effect on the parameters. Oral administration of 0.5 and 1.0 LD_50_ decreased the mean arterial blood pressure. However, no death was seen. 2.0 LD_50_ administration by intraperitoneal or oral routes suddenly decreased the mean arterial blood pressure, and the animals died, showing that at sublethal doses, DRDE-07 is safe on the cardio-vascular system [27].

### 4.3 Effect of DRDE-07 on respiratory parameters in unanesthetised rats

The respiratory effects of DRDE-07 were studied on unanesthetised rats using a body plethysmograph. Various doses of 0.5, 1.0 and 2.0 LD_50_ were given orally, and respiratory frequency and tidal volume were monitored. The animals showed a uniform breathing pattern, even at the highest dose tested, indicating that DRDE-07, even at a lethal dose, may not significantly affect respiration [28].

### 4.4 Mutagenic effect of DRDE-07 and analogues

The analogues were evaluated for mutagenic activity by the Ames Salmonella/microsome *in vitro* assay. DRDE-07 showed mutagenic activity in the TA104 strain in the absence of the S9 fraction. DRDE-30 showed mutagenic effect in the TA100 strain. DRDE-35 did not show mutagenic activity in any of the five tester strains tested [29].

Most of the studies were carried out using DRDE-07, as it was the first effective molecule identified in this series, followed by DRDE-30 and DRDE-35. As many as 50 compounds were synthesised, including known effective drugs coded in this series (hence referred to as DRDE-07 analogues), with the objective of increasing protective efficacy. The synthesis, pre-clinical studies, and initial publications in open literature took about 5 years. Considering ease of synthesis, stability, safety, and efficacy, few compounds only have been reported in the literature.

## 5 Pharmacokinetics of DRDE-07

Among the analogues, pharmacokinetic studies of DRDE-07 were carried out. A high-performance liquid chromatography (HPLC) procedure was validated for the detection of DRDE-07 and its metabolites in plasma and urine using UV absorption [30], [31]. Following oral administration, DRDE-07 appeared rapidly in plasma with a peak plasma level (C_max_) in less than 15 min. The plasma protein-bound DRDE-07 was found to be less than 25 %. The clearance of DRDE-07 was also found to be quick. Phenyl-S-ethyl amine was identified as one of the metabolites of DRDE-07 [32]. Other compounds’ pharmacokinetic studies were not reported.

## 6 Protective effect of DRDE-07 series compounds

### 6.1 Protective effect of DRDE-07 series compounds against sulphur mustard

#### 6.1.1 Protection on SM induced lethality

Amifostine is generally effective only when given by parenteral route and as a pretreatment. DRDE-07 analogues are effective when administered orally as pre-treatment or post-treatment up to 1 hr. The protection afforded by DRDE series compounds was significantly better than that of amifostine, regardless of whether it was administered intraperitoneally or orally [15], [24], [33], [34], [35]. The DRDE series compounds showed better protection than N-acetylcysteine, melatonin and sodium thiosulphate. Among DRDE series compounds, DRDE-30 showed the best protection [36], [37]. Table 3 presents the protective efficacy of the compounds, expressed as the protection index (LD_50_ with the compound divided by LD_50_ without the compound). Interestingly, similar to mice and rats, in the rabbit also, the percutaneous administration of SM was more lethal than the subcutaneous administration (p.c., 80 times more lethal) (Table 1). Oral pretreatment of DRDE-07 significantly protected HN-3 lethality by p.c. route (10.9 times), but not by s.c. route (Table 3) [9]. Many chemical decontaminants and antidotes have been experimented for SM and NM protection [5]. Among them, the DRDE series showed excellent protection as a prophylactic antidote.

**Table 3:** Protection index (PI) of DRDE series compounds.

S.No.	Agents (route)	Analogues (route)	Animal	PI
1	SM (p.c.)	DRDE-07 (p.o.)	Mice	22.8
	SM (p.c.)	DRDE-09 (p.o.)	Mice	17.1
	SM (p.c.)	DRDE-10 (p.o.)	Mice	21.5
	SM (p.c.)	DRDE-21 (p.o.)	Mice	21.5
	SM (p.c.)	DRDE-30 (p.o.)	Mice	27.1
	SM (p.c.)	DRDE-35 (p.o.)	Mice	27.1
	SM (s.c.)	DRDE-07 (p.o.)	Mice	1.2
	SM (p.o.)	DRDE-07 (p.o.)	Mice	0.7
	SM (inhalation)	DRDE-07 (p.o.)	Mice	1.0
	HN-1 (p.c.)	DRDE-07 (p.o.)	Mice	1.2
	HN-2 (p.c.)	DRDE-07 (p.o.)	Mice	1.4
	HN-3 (p.c.)	DRDE-07 (p.o.)	Mice	1.4
2	SM (p.c.)	DRDE-07 (p.o.)	Rat	2.0
	SM (p.c.)	DRDE-09 (p.o.)	Rat	2.5
	SM (p.c.)	DRDE-10 (p.o.)	Rat	2.0
	SM (p.c.)	DRDE-21 (p.o.)	Rat	2.8
	SM (p.c.)	DRDE-30 (p.o.)	Rat	2.0
	SM (p.c.)	DRDE-35 (p.o.)	Rat	1.7
3	SM (p.c.)	DRDE-07 (p.o.)	Rabbit	10.9
	HN-3 (p.c.)	DRDE-07 (p.o.)	Rabbit	1.8
	SM (s.c.)	DRDE-07 (p.o.)	Rabbit	1.0
	HN-3 (s.c.)	DRDE-07 (p.o.)	Rabbit	1.0

PI = LD_50_ with the compound/LD_50_ without the compound

SM = sulphur mustard

HN = nitrogen mustard

p.o. = per oral

p.c. = percutaneous

s.c. = subcutaneous

data sourced from [9], [16], [24], [33], [36]

#### 6.1.2 Protection against SM effect in vitro cells

SM caused leakage of lactate dehydrogenase (LDH) and alanine aminotransferase (ALT). DNA damage, loss of intracellular potassium, depletion of GSH and mitochondrial integrity were affected (MTT assay). Histopathological lesions were observed [38]. Among the various compounds studied, amifostine and DRDE-07 showed good protection, with DRDE-07 showing better results [39]. SM was cytotoxic in HeLa cells. Pretreatment of amifostine and DRDE-07 prior to SM in HeLa cells demonstrated protection against cell viability loss, as measured by the MTT assay and LDH leakage, indicating effectiveness for both compounds [40].

#### 6.1.3 Antimutagenic effect

SM induces mutagenic effect in Ames Salmonella/microsome *in vitro* assay in TA97a and TA102 strains, with or without S9 activation. Both DRDE-07 and DRDE-35 showed antimutagenic effect in the absence of S9 fraction [29].

#### 6.1.4 Protection against biochemical changes

Percutaneous exposure to SM progressively decreases the body weight of the animals (mice and rats) with a decrease in weight in all the organs, particularly in the spleen. Many of the biochemical parameters were affected, with a notable decrease in WBC, GSH, and GSSG, and an increase in serum transaminases, alkaline phosphatase, thiobarbituric acid-reactive substances, and DNA fragmentation [41]. Pretreatment with DRDE-07, DRDE-30 and DRDE-35 completely abolished all the toxic effects. Overall, DRDE-30, based on safety and protection, was better followed by DRDE-35 and DRDE-07 [17].

#### 6.1.5 Protection on pulmonary effects

SM administration causes severe pulmonary toxicity irrespective of the route [42], [43]. Dermal application of SM induces severe systemic toxicity, including the respiratory system [44]. The bronchoalveolar lavage (BALF) and lung tissues showed inflammatory cells, increased protein content, lactate dehydrogenase, myeloperoxidase, β-glucuronidase activity, matrix metalloproteases activity (MMP-2 and MMP-9) and malondialdehyde (MDA) level. There was a reduction in the GSH level and a decrease in the activities of superoxide dismutase (SOD), catalase, and glutathione-S-transferase (GST). Histopathology revealed the presence of inflammatory cells in the lung tissue [44], [45], [46]. The analogues significantly protected against all the adverse effects. DRDE-30 showed better protection compared to DRDE-07 and DRDE-35 [44]. The protective effect of the DRDE series compounds is better than that of amifostine [46]. The protective effect is due to the anti-inflammatory and antioxidant action of the compounds [47], [48].

#### 6.1.6 Protection on SM induced inflammation

Percutaneous administration of SM increased the level of the pro-inflammatory cytokine, IL-6. Histopathological evaluation showed loss of normal cellular architecture, infiltration of inflammatory cells and an increase in the number of infiltrating mast cells in the mouse skin [49]. Pretreatment with DRDE-07 ameliorated the inflammatory effect, and immunohistochemistry showed a reduced number of proliferating cell nuclear antigen (PCNA) positive cells. The anti-inflammatory effect of DRDE-07 was comparable to indomethacin and dexamethasone [50].

#### 6.1.7 Protection on monofunctional SM

The monofunctional SM (2-chloroethyl ethyl sulphide; CEES) also causes toxicity, though lesser than SM [51]. Dermal application of CEES depletes GSH and activates glutathione peroxidase and glutathione transferase. Due to oxidative stress and inflammatory effects, the proinflammatory cytokines were increased (IL-1α, IL-1β, IL-6, TNF-α, and IFN-γ). Correspondingly, the anti-inflammatory cytokines were decreased (IL-4 and IL-10). Neutrophil infiltration and increased myeloperoxidase (MPO) levels were also observed. Due to the antioxidant and anti-inflammatory effects, DRDE-07 significantly protected against the toxic effects of CEES [47], [52].

#### 6.1.8 Protection of SM induced changes on genetic pathways

Gene expression profiles carried out by global genome microarray analysis in mouse liver showed that SM exposed mice (p.c.) showed downregulation of xenobiotic cytochrome P450 pathway genes. Several other genes, including cell adhesion molecules, cytokine receptor metabolism, fatty acid metabolism, glutathione metabolism, and cell cycle signalling pathway genes, were also downregulated. All the genetic markers were normalised by oral pretreatment with DRDE-07 [53].

#### 6.1.9 Combination therapy

SM quickly forms a highly reactive sulphonium ion and attacks several macromolecules in the system. Hence, a single antidote with a specific mechanism may not be suitable for protection [5]. Combining two sulphonyl compounds, N-acetylcysteine and DRDE-07 (p.o.), showed good protection in mice as a pretreatment. The combination ameliorated the weight loss, decrease in GSH and increase in MDA induced by SM (4 LD_50_ p.c.). There was a significant reduction in the histopathological lesions induced by SM in the liver, spleen and skin [54]. Pretreatment with combinations of DRDE-07, DRDE-30, or DRDE-35, along with amifostine (p.o.), in mice could recover all the toxic effects of SM, including a decrease in body weight and organ weight, a decrease in hepatic GSH and GSSG content, and histopathological changes. The combination treatment with amifostine decreased the dose of the DRDE analogues [55].

Combination therapy or hybrid drugs would be very effective for complex and resistant diseases, including cancer [56].

### 6.2 Protection against nitrogen mustards

Nitrogen mustards (HN-1, HN-2 and HN-3) are alkylating and blister-inducing chemical warfare agents [12]. HN-2 is also known as mechlorethamine and is used as an anticancer drug [41]. The survival of mice was evaluated with the pretreatment of DRDE-07 (p.o.) against the nitrogen mustards (p.c.). DRDE-07 gave marginal protection (Table 3) [36]. Further studies were carried out with the nitrogen mustards on the biochemical effects of the important analogues. The nitrogen mustards caused a decrease in spleen weight, WBC, liver GSH and GSSG. There was an increase in liver MDA and DNA damage [17]. Treatment with DRDE-07, DRDE-30 and DRDE-35 significantly protected against the changes in spleen weight, WBC count, GSH, GSSG, MDA, and DNA damage. The protection of DRDE-07, DRDE-30 and DRDE-35 was only partial and was more or less similar [57]. Percutaneous exposure to the nitrogen mustards caused granulovacuolar degeneration and perinuclear clumping of cytoplasm in hepatocytes. The splenic lesions were congestion and hypocellularity of the white pulp, indicative of oxidative stress. All three compounds partially protected the histopathological lesions [37]. Overall, the protection of the analogues is – SM = DRDE-30 > DRDE-35 > DRDE-07 > DRDE-10; HN-1 = DRDE-30 > DRDE-10 > DRDE-35 > DRDE-07; HN-2 = DRDE-30 > DRDE-35 > DRDE-07 > DRDE-10; HN-3 = DRDE-30 > DRDE-35 > DRDE-07 > DRDE-10 (Table 4). The protection of DRDE-07 on HN-3 induced lethality was studied in rabbits. Interestingly, in the rabbit, the percutaneous administration of HN-3 was also more lethal than the subcutaneous administration (p.c., 3.3 times more lethal) (Table 1). Oral pretreatment of DRDE-07 significantly protected HN-3 lethality by p.c. route (1.8 times), but not by s.c. route (Table 3) [9].

**Table 4:** Protection by analogues on reduced glutathione (GSH), malondialdehyde (MDA) and DNA fragmentation in the liver of mice.

S.No.	Agents (p.c.)	Analogues (p.o.)	GSH % Red	MDA fold Inc	DNA fold Inc
1	Control	-	0	1	1
	SM	-	41.9	1.5	2.9
	SM	DRDE-07	4.7	1.1	1.2
	SM	DRDE-10	+ 4.7*	1.1	1.9
	SM	DRDE-30	9.3	1.1	1.1
	SM	DRDE-35	+ 11.6*	1.1	1.6
2	Control	-	0	1	1
	HN-1	-	51.2	1.8	3.3
	HN-1	DRDE-07	14.0	1.7	2.7
	HN-1	DRDE-10	16.3	1.6	2.3
	HN-1	DRDE-30	11.6	1.4	2.4
	HN-1	DRDE-35	27.9	1.6	2.7
3	Control	-	0	1	1
	HN-2	-	58.1	1.6	3.7
	HN-2	DRDE-07	65.1	1.4	3.1
	HN-2	DRDE-10	60.5	1.6	3.3
	HN-2	DRDE-30	34.9	1.4	2.9
	HN-2	DRDE-35	62.8	1.5	2.9
4	Control	-	0	1	1
	HN-3	-	74.4	2.1	4
	HN-3	DRDE-07	55.8	1.5	3.3
	HN-3	DRDE-10	62.8	1.5	3.3
	HN-3	DRDE-30	46.5	1.0	2.8
	HN-3	DRDE-35	51.2	1.3	3.2

SM = Sulphur mustard

HN-1, HN-2 and HN-3 = Nitrogen mustards

GSH = Reduced glutathione

MDA = Malondialdehyde

% Red = Percent reduction

* = Percent increase

Fold Inc = Fold increase

p.o. = per oral

p.c. = percutaneous

data sourced from [16], [17], [57]

### 6.3 Protection against radiation

Radiation and radioactive materials initiate oxidative stress and damage multiple organs. Radiotherapy for thoracic neoplasms and exposure to radioactive materials causes pneumonitis and lung fibrosis [58]. Thoracic irradiation (13.5 Gy) in C57BL/6 mice caused parenchymal opacity, a decrease in GSH, superoxide dismutase and catalase activity, and an increase in MDA, indicating oxidative stress [59]. There was apoptotic and mitotic cell death in the lung, leucocyte infiltration, activation of pro-inflammatory markers (NF-κB and p38/MAPK), release of pro-inflammatory cytokines (IL-1β; TNF-α; IL-6), up-regulation of cell adhesion molecules on the endothelial cell surface and an increase in hydroxyproline content due to the development of lung fibrosis. Pretreatment (i.p.) with DRDE-07, DRDE-30 and DRDE-35 improved the survival of the irradiated mice (DRDE-07 < DRDE-30 = DRDE-35). DRDE-30 is partially protected against all the changes caused by irradiation [59]. Ionising radiation damages hematopoietic and gastrointestinal systems. Amifostine is an established drug that protects normal tissues against ionising radiation and chemotherapeutic drugs, and is effective when administered as pretreatment and only by the parenteral route [60]. Whole body irradiated mice showed serious toxic effects with mortality. DRDE-07, DRDE-30 and DRDE-35 showed protection when given as pretreatment either by p.o. or by i.p. No animal survived in the radiation group 35 days after exposure. Orally given analogues showed protection in the order: DRDE-35 > DRDE-07 > DRDE-30. When given i.p., the order of protection was DRDE-07 > DRDE-30 > DRDE-35. The compounds decreased radiation sickness (loss of weight). There was significant protection of hematopoietic damage (peripheral blood counts and endogenous spleen colony-forming units) and gastrointestinal injury (structural integrity, villi height, regeneration of crypts and mitotic figures) [61].

### 6.4 Protection against anticancer agents

Bleomycin is one of the effective anticancer agents used for the management of several malignancies. It is known to cause pulmonary toxicity with pneumonitis and fibrosis [62]. Amifostine has been found to protect the lungs from the toxicity of bleomycin and is effective when given parenterally [63]. Since DRDE-30 has been shown to be effective orally, a study was carried out in C57BL/6 mice to evaluate the bleomycin-induced lung injury. Whole body micro-computed tomography revealed changes in lung density and lung injury, which were protected by DRDE-30. Bleomycin administration induced oxidative stress, endothelial barrier dysfunction, an increase in myeloperoxidase activity, and the release of pro-inflammatory cytokines. All these effects were suppressed by DRDE-30. In addition, DRDE-30 showed an antifibrotic effect by reducing TGF-β levels, hydroxyproline levels, and the expression of the mesenchymal marker α-smooth muscle actin [64]. DRDE-30 could be a potential drug for alleviating the side effects of bleomycin [64].

### 6.5 Protection against other toxic compounds

Abrin is a glycoprotein from the seeds of *Abrus precatorius* and is extremely toxic [65]. No effective antidote has been identified [66]. Several antidotes were screened for the potential to protect against abrin toxicity in Jurkat T lymphocytes. Based on MTT assay (dimethylthiazol-diphenyltetrazolium bromide) and neutral red cell viability assays, DRDE-07 showed partial protection similar to amifostine [67].

## 7 Conclusion

Several antidotes and medical countermeasures have been experimented with for SM, but satisfactory protection has yet to be achieved. The DRDE-07 series of compounds showed good protection with safety in pre-clinical studies as a prophylactic agent when administered orally, compared to other experimented drugs. Though many studies focused on the DRDE-07 molecule, the DRDE-30 and DRDE-35 showed better safety and protection. Among them, DRDE-30 showed the best protection for SM and nitrogen mustards against radiation, as well as serving as a cytoprotectant for anticancer agents. If any of these molecules, DRDE-07, DRDE-30, or DRDE-35, is recommended as an oral prophylactic drug, further development would occur to create a more effective broad-spectrum cytoprotectant, either as a single drug or in combination. A detailed pre-clinical study, followed by human trials to assess safety, is necessary to progress in this area.

## Funding

There is no financial support.

## Declaration of Competing Interest

The authors declare that they have no known competing financial interests or personal relationships that could have appeared to influence the work reported in this paper.

## Data Availability

No data was used for the research described in the article.
